# The complete mitochondrial genome of the important mycoparasite *Clonostachys rosea* (Hypocreales, Ascomycota)

**DOI:** 10.1080/23802359.2017.1303344

**Published:** 2017-03-30

**Authors:** Xin-Cun Wang, Zhao-Qing Zeng, Wen-Ying Zhuang

**Affiliations:** State Key Laboratory of Mycology, Institute of Microbiology, Chinese Academy of Sciences, Beijing, P.R. China

**Keywords:** Biological control agent, bionectriaceae, mitogenome, next-generation sequencing, phylogeny

## Abstract

The complete nucleotide sequence of mitochondrial genome of the important mycoparasitic fungus *Clonostachys rosea* was determined using the next-generation sequencing technology. The circular molecule is 40,921 bp long with a GC content of 27.90%. Gene prediction revealed 42 genes encoding 15 conserved proteins, 25 tRNAs, the large and small ribosomal RNAs. All genes are located on the same strand. It is found to be similar to the previously sequenced mitochondrial genomes of *Acremonium chrysogenum* and *Nectria cinnabarina*. The differences lie in the copy number of *trnG-UCC* and locations of *trnN-GUU* and *cox2*. The phylogenetic analysis confirmed *C. rosea* as a sister taxon of *A. chrysogenum*in (Bionectriaceae). The mitochondrial genome of *C. rosea* will contribute to the understanding of phylogeny and evolution of Hypocreales.

*Clonostachys rosea* is a well-known mycoparasitic fungus used as biological control agent against several important plant pathogens, including *Bipolaris sorokiana*, *Alternaria* spp. and *Fusarium* spp. (Kosawang et al. 2014). Its nuclear genome and transcriptome analyses have been reported (Kosawang et al. 2014; Karlsson et al. 2015). The fungus is economically important and widely distributed in tropical and temperate zones. In China, it has been found from 10 provinces (Zhuang 2013).

The *C. rosea* strain 6792 was isolated from an ascoma living on bark from the Tianmu Mountain (N30°18′, E119°31′), Zhejiang Province, China. The specimen was deposited in the Mycological Herbarium, Institute of Microbiology, Chinese Academy of Sciences, Beijing, China (HMAS 183484). DNA extraction, library construction, and sequencing were performed as described in the previous studies (Wang et al. 2016a, 2016b, 2016c). The 125 bp pair-end reads were assembled using CLC Genomics Workbench (Version 8.0.3, CLC Bio, Aarhus, Denmark). The mitochondrial genome (mitogenome) of *Trichoderma reesei* (NC_003388) was served as reference to identify the assembled scaffolds belonging to mitogenome. After filtering with BLAST, only one scaffold was obtained. Manual comparison of the ends of the scaffold helped to link them into a circular molecule. This mitogenome was annotated using MFannot (Lang et al. 2014). Nineteen mitogenomes belonging to Hypocreales and one outgroup in Sordariales were included in the Neighbor-Joining phylogenetic analysis using MEGA6 (Tamura et al. 2013).

The complete sequence of *C. rosea* mitogenome (GenBank accession number KU668563) is 40,921 bp long with the GC content of 27.90%. It encodes 15 conserved proteins, 25 tRNAs, and the large and small ribosomal RNAs. All structural genes are located on the same strand. The tRNA genes contain codons for all 20 standard amino acids. Most amino acids are represented by only one tRNA gene; however, two *trnL* (*trnL-UAA* and *trnL-UAG*), two *trnR* (*trnR-ACG* and *trnR-UCU*), two *trnS* (*trnS-GCU* and *trnS-UGA*), and three *trnM-CAU* genes are found in this mitogenome. Eight introns are detected in five genes, i.e. *rnl* (1), *nad5* (1), *cob* (1), *cox1* (4), and *nad1* (1).

Mitogenomes of the two members in Bionectriaceae, *C. rosea* and *Acremonium chrysogenum* (NC_023268), share the same composition and order of protein, rRNA and tRNA genes except the location of *trnN-GUU* and an addition of *trnG-UCC* for the latter. Compared with the mitogenome of *Nectria cinnabarina*, the representative of Nectriaceae, the quantity and order of their protein, rRNA and tRNA genes are identical except the location of *cox2*.

As shown in Figure 1, five monophyletic clades, correlation to Bionectriaceae, Clavicipitaceae, Cordycipitaceae, Hypocreaceae, and Nectriaceae, are well resolved in the phylogenetic analysis. *Clonostachys rosea* and *A. chrysogenum* are sisters in Bionectriaceae. This family, clustered with *Acremonium implicatum*, islocated at the basal position of Hypocreales. This is in accordance with the combined five nuclear gene analysis (Sung et al. 2007). The mitogenome of *C. rosea* will contribute to the understanding of phylogeny and evolution of Hypocreales.

**Figure 1.**
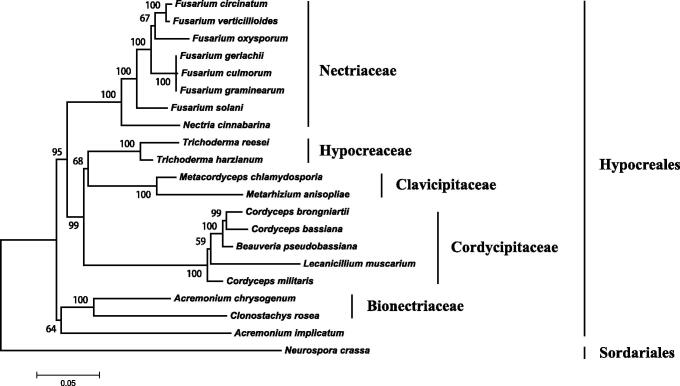
Phylogenetic relationship of 20 taxa of Hypocreales (Ascomycota) determined by neighbor-joining analysis based on concatenated sequences of 15 translated mitochondrial proteins. The 15 proteins included subunits of the respiratory chain complexes (*cob, cox1*, *cox2*, *cox3*), ATPase subunits (*atp6*, *atp8*, and *atp9*), NADH: quinone reductase subunits (*nad1*, *nad2*, *nad3*, *nad4*, *nad4L*, *nad5*, *nad6*), and ribosomal protein S3 (*rps3*). The concatenated sequences were aligned using MAFFT. The following 19 mitogenomes were used in this analysis: *Acremonium chrysogenum* (NC_023268), *A. implicatum* (NC_026534), *Beauveria pseudobassiana* (NC_022708), *Cordyceps bassiana* (NC_010652), *C. brongniartii* (NC_011194), *C. militaris* (NC_022834), *Fusarium circinatum* (NC_022681), *F. culmorum* (NC_026993), *F. gerlachii* (NC_025928), *F. graminearum* (NC_009493), *F. oxysporum* (NC_017930), *F. solani* (NC_016680), *F. verticillioides* (NC_016687), *Lecanicillium muscarium* (NC_004514), *Metacordyceps chlamydosporia* (NC_022835), *Metarhizium anisopliae* (NC_008068), *Nectria cinnabarina* (KT731105), *Trichoderma harzianum* (KR952346) and *T. reesei* (NC_003388). *Neurospora crassa* (NC_026614) was served as an outgroup. The percentages of replicate trees in which the associated taxa clustered together in the bootstrap test (1000 replicates) are shown next to the branches.
